# Concurrent Types of Intracranial Hemorrhage are Associated with a Higher Mortality Rate in Adult Patients with Traumatic Subarachnoid Hemorrhage: A Cross-Sectional Retrospective Study

**DOI:** 10.3390/ijerph16234787

**Published:** 2019-11-29

**Authors:** Cheng-Shyuan Rau, Shao-Chun Wu, Shiun-Yuan Hsu, Hang-Tsung Liu, Chun-Ying Huang, Ting-Min Hsieh, Sheng-En Chou, Wei-Ti Su, Yueh-Wei Liu, Ching-Hua Hsieh

**Affiliations:** 1 Department of Neurosurgery, Kaohsiung Chang Gung Memorial Hospital, Chang Gung University and College of Medicine, Kaohsiung 83301, Taiwan; ersh2127@cloud.cgmh.org.tw; 2 Department of Anesthesiology, Kaohsiung Chang Gung Memorial Hospital, Chang Gung University and College of Medicine, Kaohsiung 83301, Taiwan; shaochunwu@gmail.com; 3 Department of Trauma Surgery, Kaohsiung Chang Gung Memorial Hospital, Chang Gung University and College of Medicine, Kaohsiung 83301, Taiwan; ah.lucy@hotmail.com (S.-Y.H.); htl1688@yahoo.com.tw (H.-T.L.); junyinhaung@yahoo.com.tw (C.-Y.H.); hs168hs168@gmail.com (T.-M.H.); athenechou@gmail.com (S.-E.C.); s101132@adm.cgmh.org.tw (W.-T.S.); 4 Department of General Gurgery, Kaohsiung Chang Gung Memorial Hospital, Chang Gung University and College of Medicine, Kaohsiung 83301, Taiwan; 5 Department of Plastic Surgery, Kaohsiung Chang Gung Memorial Hospital, Chang Gung University and College of Medicine, Kaohsiung 83301, Taiwan

**Keywords:** traumatic brain injury, subarachnoid hemorrhage (SAH), subdural hematoma (SDH), epidural hematoma (EDH), intracerebral hemorrhage (ICH), mortality

## Abstract

Traumatic subarachnoid hemorrhage (SAH) is the second most frequent intracranial hemorrhage and a common radiologic finding in computed tomography. This study aimed to estimate the risk of mortality in adult trauma patients with traumatic SAH concurrent with other types of intracranial hemorrhage, such as subdural hematoma (SDH), epidural hematoma (EDH), and intracerebral hemorrhage (ICH), compared to the risk in patients with isolated traumatic SAH. We searched our hospital’s trauma database from 1 January, 2009 to 31 December, 2018 to identify hospitalized adult patients ≥20 years old who presented with a trauma abbreviated injury scale (AIS) of ≥3 in the head region. Polytrauma patients with an AIS of ≥3 in any other region of the body were excluded. A total of 1856 patients who had SAH were allocated into four exclusive groups: (Group I) isolated traumatic SAH, *n* = 788; (Group II) SAH and one diagnosis, *n* = 509; (Group III) SAH and two diagnoses, *n* = 493; and (Group IV) SAH and three diagnoses, *n* = 66. One, two, and three diagnoses indicated occurrences of one, two, or three other types of intracranial hemorrhage (SDH, EDH, or ICH). The adjusted odds ratio with a 95% confidence interval (CI) of the level of mortality was calculated with logistic regression, controlling for sex, age, and pre-existing comorbidities. Patients with isolated traumatic SAH had a lower rate of mortality (1.8%) compared to the other three groups (Group II: 7.9%, Group III: 12.4%, and Group IV: 27.3%, all *p* < 0.001). When controlling for sex, age, and pre-existing comorbidities, we found that Group II, Group III, and Group IV patients had a 4.0 (95% CI 2.4–6.5), 8.9 (95% CI 4.8–16.5), and 21.1 (95% CI 9.4–47.7) times higher adjusted odds ratio for mortality, respectively, than the patients with isolated traumatic SAH. In this study, we demonstrated that compared to patients with isolated traumatic SAH, traumatic SAH patients with concurrent types of intracranial hemorrhage have a higher adjusted odds ratio for mortality.

## 1. Introduction

Traumatic brain injury (TBI) is a leading cause of death in trauma patients. In patients with TBI, the traumatic subarachnoid hemorrhage (SAH) is the second-most frequent intracranial hemorrhage [1] and a common radiologic finding on computed tomography (CT) [2]. It is estimated that in patients with moderate or severe TBI, 33–60% have traumatic SAH [3,4,5,6]. Furthermore, the diagnosis of traumatic SAH continues to increase with the improved resolution of newer-generation CT scan machines [7].

The actual death rate and the requirement for neurosurgical intervention in SAH cases is significantly lower than in cases of non-SAH intracranial hemorrhages [8]. In addition, the outcome is good for patients with isolated traumatic SAH, which is defined as the exclusive presence of SAH without any other traumatic, radiographic intracranial pathology in the trauma patient. In isolated traumatic SAH patients with mild TBI and a Glasgow Coma Scale (GCS) score ≥13, neurologic deterioration or a requirement for neurosurgical intervention was rarely reported [2,9,10,11,12]. A meta-analysis study revealed that in patients with isolated trauma SAH, the cumulative incidences of radiographic progression were 5.76% (95% confidence interval [CI] 1.18–12.9%) and the incidences of neurosurgical procedures were 0.0017% (95% CI 0–0.39%) [13]. Furthermore, the meta-analysis of eight studies, which included a total of 873 isolated traumatic SAH patients, reported a mortality rate ranging from 0–2.5% [13]. Some studies advocated that the presence of isolated traumatic SAH does not warrant repeat CT imaging, admission into the ICU, or a transfer to a tertiary referral center [2,14,15,16].

In contrast to the good prognosis for patients with isolated traumatic SAH, the outcome is less positive for patients who have traumatic SAH in the presence of other intracranial hemorrhages [13,17]. Traumatic SAH indicates the existence of greater mechanical forces and intracranial deformation during the initial injury [18]. The presence of traumatic SAH in cerebral contusion is an independent predictor for the progression of hematoma [19,20,21]. In 20% of patients with severe TBI, symptomatic cerebral vasospasm may develop together with traumatic SAH [22,23]. However, the mortality odds for patients with other concurrent intracranial hemorrhages have been explored less in the literature than those for isolated traumatic SAH. Therefore, by controlling for baseline differences in patient characteristics such as sex, age, and pre-existing comorbidities, this study aimed to estimate the risk of mortality in adult trauma patients with traumatic SAH and concurrent intracranial hemorrhages compared to the risk in patients with isolated traumatic SAH.

## 2. Materials and Methods

### 2.1. Ethics Statement

This study was approved by the institutional review board (IRB) of the Kaohsiung Chang Gung Memorial Hospital, a level I trauma center in southern Taiwan (approval number 201901261B0). Given that this is a retrospective study using the registered trauma database of the hospital [24,25,26], the requirement for obtaining informed consent from the patients was waived.

### 2.2. Study Population

**Figure 1:**
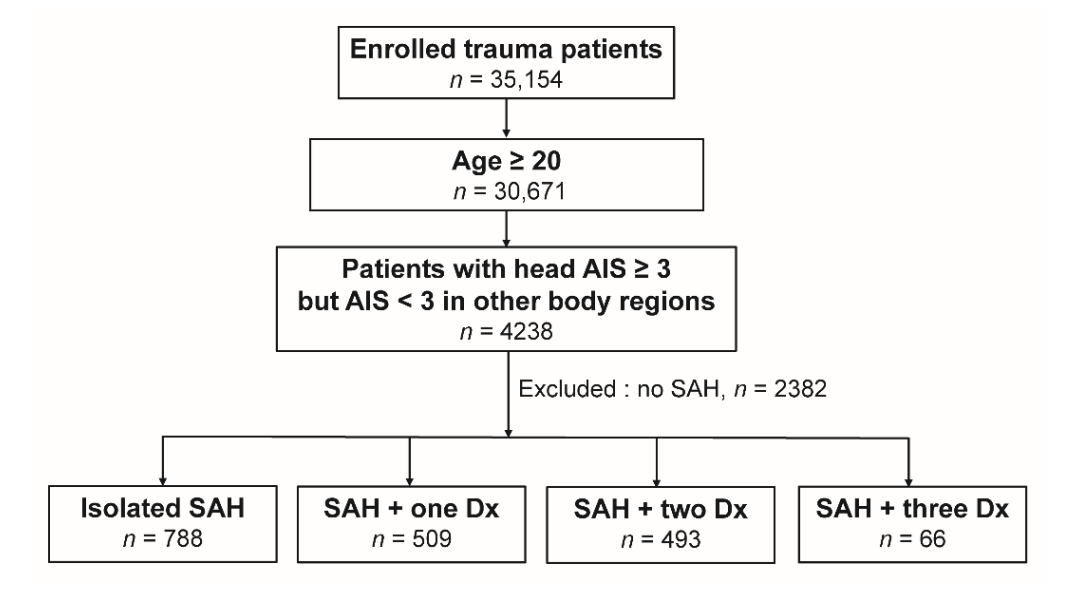
Flow chart illustrating the inclusion of adult patients with acute traumatic subarachnoid hemorrhage and the allocation of these patients into four groups. AIS—abbreviated injury scale, Dx—diagnosis, and SAH—subarachnoid hemorrhage. One, two, and three diagnoses indicated occurrences of one, two, or three other types of intracranial hemorrhage (subdural hematoma, epidural hematoma, or intracerebral hemorrhage).

We searched the 10-year span between 1 January, 2009 and 31 December, 2018 in the registered trauma database to identify hospitalized adult patients ≥20 years old who presented with a trauma abbreviated injury scale (AIS) of ≥3 in the head region. To avoid the effect of fatal injuries to other body regions as confounding factors on the assessment of mortality, we excluded polytrauma patients with an AIS of ≥3 in any other region of the body [27]. The enrolled patients were identified using the diagnostic injury codes 852.0 and 852.1 (traumatic subarachnoid hemorrhage), 852.2 and 852.3 (subdural hematoma), 852.4 and 852.5 (epidural hematoma), and 853 (intracerebral hemorrhage), from the *International Classification of Diseases, Ninth Revision, Clinical Modification* (ICD-9-CM), and those patients without acute traumatic SAH and without brain hemorrhage were not included in this study. Thus, the included patients (Figure 1) were allocated into four groups: (1) isolated traumatic SAH, (2) SAH and one diagnosis, (3) SAH and two diagnoses, and (4) SAH and three diagnoses. One diagnosis indicated an occurrence of one other type of intracranial hemorrhage, such as subdural hematoma (SDH), epidural hematoma (EDH), or intracerebral hemorrhage (ICH); two and three diagnoses indicated occurrences of two or three other types of intracranial hemorrhage, respectively. The retrieved patient information included sex; age; comorbidities including hypertension (HTN), diabetes mellitus (DM), coronary artery disease (CAD), congestive heart failure (CHF), cerebral vascular accident (CVA), and end-stage renal disease (ESRD); Glasgow Coma Scale; injury severity score (ISS); length of stay (LOS) in hospital; and in-hospital mortality. The comorbidities of the patients were validated and recorded in the registered trauma database during the time of hospitalization. The in-hospital mortality by all causes (e.g., direct effect from the intracranial hemorrhage or associated morbidities like multiple organs failure, shock, sepsis, acute respiratory distress, pneumonia, and renal failure) was recorded in the registered trauma database during the time of hospitalization. In the registered trauma database, the patients who were declared dead at the scene of the accident were not recorded as in-hospital mortalities and no information regarding one-month or one-year mortality was collected. For fatal patients, the length of stay in hospital ended in the event of death. Those subjects with incomplete registered data were excluded from the study.

### 2.3. Statistical Analysis

The statistical analysis was performed using SPSS 23.0 Windows software (IBM Corp., Armonk, NY, USA). The homogeneity of variance of the continuous variables was first assessed using Levene’s test, followed by a one-way analysis of variance (ANOVA) with a Games–Howell posthoc test, which was used to evaluate the differences among the allocated groups of patients. We expressed the continuous data in mean ± standard deviation, while presenting the GCS and ISS as a median and interquartile range (IQR, Q1–Q3), respectively. The in-hospital mortality of patients was defined as the primary outcome of this study. The odds ratios (ORs) of mortality were calculated with a 95% confidence interval. The adjusted odds ratio (AOR) of mortality was calculated with logistic regression, controlling for sex, age, and pre-existing comorbidities. The difference was considered significant when a *p* value of <0.05 was obtained.

## 3. Results

### 3.1. Characteristics of the Patients

**Figure 2:**
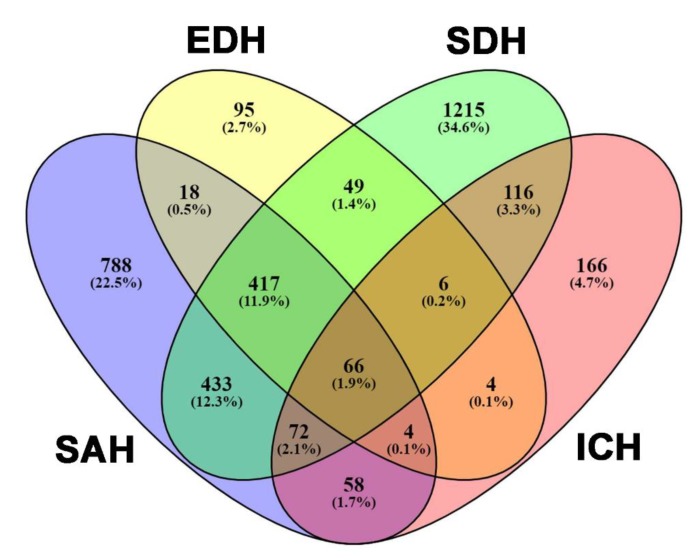
The allocation of adult patients with acute traumatic subarachnoid hemorrhage into four groups: SAH, epidural hematoma (EDH), subdural hematoma (SDH), and intracerebral hemorrhage (ICH).

In this study, we identified a total of 4238 adult trauma patients with a traumatic brain injury and an AIS of ≥3 in the head region and <3 in other body regions. After excluding those without SAH (*n* = 2382) and those with incomplete registered data (*n* = 0), 1856 patients with SAH were allocated into four exclusive groups: (Group I) isolated traumatic SAH, *n* = 788; (Group II) SAH and one diagnosis, *n* = 509; (Group III) SAH and two diagnoses, *n* = 493; and (Group IV) SAH and three diagnoses, *n* = 66. In addition, among the 4238 patients, 3507 had at least one type of intracranial hemorrhage such as SAH, EDH, SDH, or ICH (Figure 2). Among the types of intracranial hemorrhages, SDH (67.7%) and SAH (52.9%) presented as the first and second most frequently encountered intracranial hemorrhages, respectively.

As shown in Table 1, compared with the other groups of patients, there were significantly fewer female patients in the isolated traumatic SAH group (Group I) and these patients were significantly younger. Regarding pre-existing comorbidities, the prevalence of pre-existing HTN, DM, and CVA was significantly different among these groups. Compared to Group I patients, there were significantly more patients in Group III who had HTN and DM. The group of patients with isolated traumatic SAH had a significantly higher GCS score (median [Q1–Q3]: 15 [14,15]) than the other three groups (Group II: 14 [10,11,12,13,14,15], Group III: 13 [7,8,9,10,11,12,13,14,15], and Group IV: 8 [4,5,6,7,8,9,10,11,12,13,14]), as well as a smaller percentage of patients with a GCS score of ≤ 8 and a greater percentage of patients with a GCS score of ≥13. In addition, the group of patients with isolated traumatic SAH had a significantly lower ISS (median [Q1–Q3]: 11 [9,10,11,12,13,14]) than the other three groups (Group II: 16 [16,17,18,19,20], Group III: 17 [16,17,18,19,20,21,22,23,24], and Group IV: 21 [16,17,18,19,20,21,22,23,24,25]) and presented with a greater percentage of patients with a ISS of <16 and a smaller percentage of patients with a ISS between 16–24 and ≥25. The group of patients with isolated traumatic SAH received a significantly lower rate of craniotomy (4.4%) than the other three groups (Group II: 17.3%, Group III: 33.3%, and Group IV: 36.4%)

### 3.2. Patient Outcome

The hospital LOS (8.6 days) of the patients with isolated traumatic SAH was significantly shorter than the LOS of the other three groups (Group II: 12.7 days, Group III: 13.7 days, and Group IV: 15.9 days, all *p* < 0.001). Patients with isolated traumatic SAH had a lower rate of mortality (1.8%) compared to the other three groups (Group II: 7.9%, Group III: 12.4%, and Group IV: 27.3%, all *p* < 0.001). Therefore, the odds ratio for mortality for the patients in Group II, Group III, and Group IV was 4.7 (95% CI 2.5–8.8), 7.8 (95% CI 4.3–14.1), and 20.7 (95% CI 9.7–44.2) times higher, respectively, than it was for the patients with isolated traumatic SAH. In addition, Group III patients had a significantly higher odds ratio for mortality (OR 1.7, 95% CI 1.1–2.5, *p* = 0.018) than the Group II patients, and the Group IV patients had a significantly higher odds ratio for mortality (OR 2.7, 95% CI 1.5–4.5, *p* = 0.001) than the Group III patients. Notably, no mortality was found in isolated traumatic SAH patients with mild TBI (GCS ≥13). After adjusting for sex, age, and pre-existing comorbidities, the results were similar. The adjusted odds ratio for mortality for patients in Group II, Group III, and Group IV was 4.0 (95% CI 2.4–6.5), 8.9 (95% CI 4.8–16.5), and 21.1 (95% CI 9.4–47.7)-times higher, respectively, than it was for the patients with isolated traumatic SAH. In addition, Group III patients had a significantly higher adjusted odds ratio for mortality (AOR 2, 95% CI 1.2–3.0, *p* = 0.004) than the Group II patients. Group IV patients had a significantly higher odds ratio for mortality (AOR 2.6, 95% CI 1.4–4.8, *p* = 0.003) than the Group III patients.

## 4. Discussion

In this study, we found that in the presence of traumatic SAH, there is a higher mortality rate in patients with concurrent diagnoses of other intracranial hemorrhages. When gender and pre-existing comorbidities were considered, the adjusted odds ratio of mortality for the patients in Group II, Group III, and Group IV was approximately 4, 9, and 21 times higher, respectively, than it was for the patients with an isolated traumatic SAH. In addition, the LOS in the hospital for patients with isolated SAH was significantly longer when these patients had a concurrent diagnosis of another intracranial hemorrhage.

Due to the unpredictable clinical course and the different associated severities of illness, caring for patients with traumatic SAH presents unique challenges [28]. Traumatic SAH-induced vasospasm, hydrocephalus, and pituitary or hypothalamic dysfunction may be causes of poor outcomes in these patients [6,21,29,30]. As shown in this study, the presence of subarachnoid blood associated with another type of intracranial hemorrhage indicates a poor outcome. Therefore, patients who have traumatic SAH often receive intensive care in the ICU. Even for patients with mild TBI who have isolated traumatic SAH, admission into the ICU is frequent despite the low probability of neurosurgical intervention [31]. Nonetheless, some studies have suggested that critical care admission for patients with isolated traumatic SAH may not always be indicated [32,33,34]. In a study of 14,146 patients with isolated traumatic SAH from 215 trauma centers, a total of 6313 subjects (constituting 44.6% of subjects) were admitted to the ICU for critical care [31]; however, neurosurgical intervention for these patients was only 0.24% (34/14,146), with craniotomies in 27 patients and craniectomies for 2 patients [31]. In this study, patients with isolated traumatic SAH had a lower rate of mortality (1.8%) but no mortality was found in patients with mild TBI (GCS ≥ 13). Accordingly, considering that the process of transfer is costly and may lead to the saturation of tertiary care centers with neurosurgical capabilities, we agree with the recommended protocol, which suggests that for a patient with isolated traumatic SAH and a GCS score of 15, the transfer for neurosurgical consultation may be omitted [13,35].

There were some limitations to this study. First, this review is limited by the lack of randomized, controlled trials and the paucity of prospective studies. A selection bias may be encountered in the design of the retrospective analysis in this study. Second, considering that the registered trauma database excluded patients that were declared dead at the scene of the accident and only included in-hospital mortalities, and no information regarding one-month or one-year mortalities was included, a selection bias may exist in the assessment of mortality rate. Third, a bias may be caused by the fact that we did not account for the amount of blood resulting from SAH, considering that a multivariate analysis showed that the maximum thickness (mm) of traumatic SAH was independently associated with mortality [17]. The lack of thickness, and the size, of EDH, SDH, and ICH also comprised limitations in the analysis of the outcome. Furthermore, the coagulopathy status of the patients was unexplored in this study and the lack of such information may present as a confounding factor in the outcome measure. In addition, the lack of information regarding cognitive disorders, atrial fibrillation, and warfarin treatment presents as a limitation of this study, since these factors may comprise confounding factors in the outcome analysis [36]. Lastly, the indications and results of surgical interventions for these patients were not considered in this study, and we can only assume that the operations for these patients did not result in a difference in patient management. However, considering that a craniotomy can have a beneficial effect on a patient’s outcome, the estimate of mortality risk would have been higher for those traumatic SAH patients with more concurrent types of intracranial hemorrhage if no operation had been performed.

## 5. Conclusions

In this study, we demonstrated that compared to patients with an isolated traumatic SAH, patients with traumatic SAH and concurrent types of intracranial hemorrhage have a higher adjusted odds ratio of mortality.

## Figures and Tables

**Table 1:** Characteristics and outcomes of adult patients who sustained a traumatic subarachnoid hemorrhage.

Variables	Group I (Isolated SAH) *n* = 788		Group II (SAH + one Dx) *N* = 509		Group III (SAH + two Dx) *n* = 493		Group IV (SAH + three Dx) *n* = 66		*p*
Sex, *n* (%)									<0.001
Male	401	(50.9)	318	(62.5) *	359	(72.8) *	53	(80.3) *	
Female	387	(49.1)	191	(37.5) *	134	(27.2) *	13	(19.7) *	
Age (years)	55.4	±18.5	59.6	±18.4 *	49.0	±19.4 *	52.6	±18.9	<0.001
Comorbidities, *n* (%)									
HTN	236	(29.9)	182	(35.8)	114	(23.1) *	12	(18.2)	<0.001
DM	130	(16.5)	102	(20.0)	48	(9.7) *	6	(9.1)	<0.001
CAD	28	(3.6)	30	(5.9)	21	(4.3)	3	(4.5)	0.256
CHF	10	(1.3)	6	(1.2)	2	(0.4)	0	(0.0)	0.356
CVA	39	(4.9)	34	(6.4)	16	(3.2)	1	(1.5)	0.045
ESRD	8	(1.0)	14	(2.8)	9	(1.8)	0	(0.0)	0.076
GCS, median (IQR)	15	(14–15)	14	(10–15) *	13	(7–15) *	8	(4–14) *	<0.001
1–8, *n* (%)	77	(9.9)	110	(21.6) *	149	(30.2) *	37	(56.1) *	<0.001
9–12	74	(9.4)	71	(13.9)	73	(14.8) *	8	(12.1)	0.015
≥13, *n* (%)	637	(80.8)	328	(64.4) *	271	(55.0) *	21	(31.8) *	<0.001
ISS, median (IQR)	11	(9–14)	16	(16–20) *	17	(16–24) *	21	(16–25) *	<0.001
<16, *n* (%)	595	(75.5)	90	(17.7) *	66	(13.4) *	5	(7.6) *	<0.001
16–24, *n* (%)	173	(22.0)	363	(71.3) *	331	(67.1) *	37	(56.1) *	<0.001
≥25, *n* (%)	20	(2.5)	56	(11.0) *	96	(19.5) *	24	(36.4) *	<0.001
Craniotomy, *n* (%)	35	(4.4)	88	(17.3) *	164	(33.3) *	24	(36.4) *	<0.001
Hospital LOS (days)	8.6	±9.3	12.7	±13.1 *	13.7	±13.2 *	15.9	±14.3 *	<0.001
Mortality, *n* (%)	14	(1.8)	40	(7.9) *	61	(12.4) *	18	(27.3) *	<0.001
Mortality OR	-		4.7	(2.5–8.8)	7.8	(4.3–14.1)	20.7	(9.7–44.2)	<0.001
Mortality AOR	-		4.0	(2.4–6.5)	8.9	(4.8–16.5)	21.1	(9.4–47.7)	<0.001

One diagnosis indicated an occurrence of one other type of intracranial hemorrhage (SDH, EDH, or ICH); two and three diagnoses indicated occurrences of two or three other types of other intracranial hemorrhages, respectively. CAD—coronary artery disease, CHF—congestive heart failure, CI—Confidence interval, CVA—cerebral vascular accident, DM—diabetes mellitus, ESRD—end-stage renal disease, GCS—Glasgow Coma Scale, HTN—hypertension, IQR—interquartile range, ISS—injury severity score, LOS—length of stay, OR = odds ratio, and * indicates significant difference vs. Group I.
